# Shaping taxonomic and functional diversity in tropical lakes: out-lake factors set the stage across waterscapes

**DOI:** 10.1007/s11356-026-37554-w

**Published:** 2026-03-01

**Authors:** Rayanne Barros Setubal, Otávio Sena, Camila Rodrigues Cabral, Regina Lúcia Guimarães Nobre, Letícia Barbosa Quesado, Reinaldo Luiz Bozelli, Rafael Dettogni Guariento, Luciana Silva Carneiro, Adriano Caliman

**Affiliations:** 1 Universidade Federal Do Rio de Janeiro, Rio de Janeiro, RJ Brazil; 2 Centro de Biociências, Universidade Federal Do Rio Grande Do Norte, Natal, RN Brazil; 3 Universidade Federal Do Mato Grosso Do Sul, Campo Grande, MS Brazil

**Keywords:** Functional divergence, Shallow lakes, Land use, Reservoirs, Zooplankton, Functional diversity, Waterscapes

## Abstract

The biodiversity of lake ecosystems is partly shaped by a variety of local environmental factors that collectively form the lake’s waterscape. These factors range from in-lake variables, such as the structure of habitats and aquatic communities, and the physical and chemical conditions of the water, to variables related to lake geomorphology, lake compartments and history (e.g., lake morphometry, habitat identity, and origin), as well as out-lake landscape-scale factors, such as catchment and land use properties and climate. Here, we investigated the interplay of multiple in-lake and out-lake environmental factors on the functional and taxonomic diversity of zooplankton across 98 tropical lakes spanning an area of approximately 36,000 km^2^. Through a regression tree and random forest analysis, we verified the importance of several factors on the taxonomic and functional diversity of zooplankton. Lake perimeter-to-lake volume ratio, together with variables related to land use in the surrounding areas and in the catchment, were decisive for interactively determining the taxonomic and functional diversity of the zooplankton community. The high connectivity and susceptibility of the environments to external input together with high precipitation, and consequently greater input of allochthonous material into the lakes, negatively impacted zooplankton diversity. We observed that biodiversity responses to multiple in-lake and out-lake factors can be complex. Functional diversity measures were effective in detecting the effects of ultimate factors that operate at larger scales, especially out-lake factors. Taxonomic diversity measures were relevant in detecting the interactive effects of both out-lake and in-lake factors. Our findings highlight the critical role of both in-lake and out-lake factors in shaping zooplankton diversity, emphasizing the need for comprehensive management strategies that consider the multifaceted interactions encompassing the waterscape of lake ecosystems and their surrounding catchments.

**Supplementary Information:**

The online version contains supplementary material available at 10.1007/s11356-026-37554-w.

## Introduction

Understanding the underlying mechanisms associated with variation in species distribution and community structure across space and time is a fundamental goal of ecology and environmental management. In lake ecosystems, most studies disentangling the causes of local species composition and abundance focus on well-recognized local biotic and abiotic factors such as food web structure, resource availability, and limnological features (Jeppesen et al. 2010; Wiafe et al. 2008). This is especially true for planktonic communities of lakes, which show a fast response to dynamic local environmental changes (Amsinck et al. 2006; Guariento et al. 2010; Jeppesen et al. 2005). However, lake plankton is affected by a multitude of proximate and ultimate factors operating at different temporal and spatial scales across the catchment-to-lake waterscape continuum (Karpouzoglou and Vij 2017; O’Sullivan et al. 2022). These factors include both in-lake biotic and abiotic environmental characteristics, and out-lake catchment and climate properties, such as land cover-land use and precipitation. Studies investigating the relative importance of in- and out-lake environmental factors, as well as their interaction, on lake plankton are, however, scarce (but see Dodson et al. (2005, 2007); Berggren et al. (2018); Porcel et al. (2020)). Since in- and out-lake factors may influence the aquatic environment through different mechanisms, magnitudes, and temporal scales (Håkanson 2005; Staehr et al. 2012; Heino et al. 2021), this gap may challenge our ability to understand the causes of biodiversity changes in lakes and to implement effective strategies for its protection and restoration (Dos Santos et al. 2025).

Lakes are concave-recipient ecosystems dynamically connected to their catchments by the fluxes of energy and matter across their borders (Leroux and Loreau 2008). These cross-ecosystem flows have important implications for the lake’s water quality and biodiversity (Dodson et al. 2005; Leroux and Loreau 2008; Knoll et al. 2015; Nobre et al. 2020). The strength and magnitude of land-to-lake coupling may depend on factors associated with the landscape properties of lake catchments (e.g., size, topography, land use type, diversity, extent, and distance to the water body) (Declerck et al. 2006; Nobre et al. 2020) and to the lake’s geomorphology (e.g., lake type/origin, size, and perimeter) (Bremigan et al. 2008). These factors can individually, additively, or interactively influence the composition and/or the amount of allochthonous material entering lakes, as well as the relative importance of cross-ecosystem flows to the lake ecosystem functioning (Foley et al. 2005; Vanni et al. 2005; Nobre et al. 2020). For example, predictor variables that combine lakes’ landscape properties and morphometry, such as catchment size-to-lake volume ratio, may provide valuable information on the absolute and relative magnitude of external inputs of matter and may indicate the susceptibility of lakes functioning to changes in their catchments (Håkanson 2005; Bremigan et al. 2008; Doubek and Carey 2017). This is especially true for artificial lakes (i.e., reservoirs) that have on average larger catchments than natural lakes (Bengtsson et al. 2012) and, therefore, are more threatened than natural lakes to changes promoted by human use of their catchments (Doubek and Carey 2017).

In addition, land-to-lake coupling may critically depend on precipitation magnitude since intense rainfall events are expected to increase terrestrial inputs through surface runoff, erosion, and/or leaching, while in prolonged periods of drought, the runoff is reduced (Carpenter et al. 1992; Jeppesen et al. 2015). More complex scenarios are also possible since precipitation can interact with the lake’s geomorphology and landscape properties in mediating the flow of allochthonous material into lakes (Knoll et al. 2015; Nobre et al. 2020). Considering the increasing human pressure on freshwater ecosystems and their catchments for the development of anthropogenic activities, the aforementioned conjectures affecting land-to-lake coupling and, consequently, lake biodiversity, have gained relevance (Dodson et al. 2007; Burgin et al. 2016), and accumulating evidence points out to positive connections between the lake’s catchment integrity and lake’s biodiversity (Johnson and Host 2010; Doubek and Carey 2017; Medeiros et al. 2019; Tromboni et al. 2019).

Moving toward components that integrate lake features, factors such as lake type and/or origin and the type and structural complexity of lake habitats may also have important effects on their aquatic communities. For example, reservoirs are newer systems when compared to natural lakes. The geomorphological and hydrodynamic changes that occurred throughout the history of the natural lakes have subjected their biological communities to a lengthy process of adaptive selection to such conditions (Albrecht and Wilke 2008). According to the *structural heterogeneity hypothesis* (McKindsey and Bourget 2001) and *ecological time hypothesis* (Dodson et al. 2007; Mittelbach et al. 2007; Heino et al. 2021), old habitats are structurally more complex and had more time to be colonized, thus having the potential to host higher biodiversity. In addition, the horizontal compartmentalization of lake habitats, which creates distinct environmental conditions within a single ecosystem, is an additional factor capable of affecting planktonic communities, especially zooplankton. In comparison with the littoral habitat, the limnetic habitat is usually deeper with possible vertical stratification of the physicochemical variables (Schindler and Scheuerell 2002). The littoral habitat is under the direct influence of the adjacent terrestrial ecosystem which can contribute to the littoral habitat heterogeneity through the allochthonous input of leaves and branches from riparian vegetation (Carpenter and Lodge 1986; Cronin et al. 2006; Thomaz et al. 2008). Although there are considerable differences between limnetic and littoral habitats, studies have historically focused on the limnetic ones (Schindler and Scheuerell 2002), disregarding the possible role of the littoral region as an environmental filter on species and trait selection.

Zooplankton plays an essential role in the aquatic food webs, linking primary production to upper trophic levels (Soranno et al. 1993; Barnett et al. 2007; Declerck and de Senerpont Domis 2023). It constitutes a diverse and abundant group, with species that vary greatly in size, ontogenetic development, growth, reproductive and feeding strategies, and trophic levels (Figuerola and Green 2002; Hébert et al. 2016). The high diversity of trait variability, dispersal capacity, and the fast generation time of zooplankton species makes them extremely sensitive to changes in the aquatic environment across space and time (Barnett et al. 2007; Hébert et al. 2017) and, consequently, an efficient bioindicator of environmental changes (Walseng et al. 2006; Dodson et al. 2007). Despite their relevance, there are major gaps in our knowledge about zooplankton community structure across freshwater tropical ecosystems (Merrix-Jones et al. 2013). One of these gaps is the lack of studies focusing on understanding zooplankton biodiversity patterns and their underlying mechanisms through a trait-based approach (Beisner et al. 2006; but see Braghin et al. (2018) and Setubal et al. (2020)). Compared to the traditional taxonomic approach, the use of trait-based approaches in community ecology has the potential to better explain and predict functional community structure and dynamics in response to environmental gradients (Lavorel et al. 2008; Litchman et al. 2013). On the other hand, the combined use of both taxonomic and trait-based approaches has proven promising in studies designed to disentangle the mechanisms affecting community structure. This approach provides complementary answers and insights, allowing us to detect and infer the causes of biodiversity patterns (Longhi and Beisner 2010; Jarzyna and Jetz 2018).

Here, our objective was to investigate whether and how patterns of zooplankton taxonomic and functional diversity vary in function of a set of in-lake and out-lake variables. Predictor variables encompassed the entire catchment-to-lake waterscape and ranged from integrative variables which are broadly descriptors of climatic, physical, geological, and anthropogenic characteristics of the ecosystems, such as land use properties, precipitation, lake origin (natural or artificial), and habitat identity (pelagic or littoral), to more local proximate limnological variables which are more punctual descriptors of the water environment. We hypothesize that out-lake predictors related to mechanisms operating on broader temporal scales, such as those related to the lake’s age, and those integrating the interaction between the aquatic ecosystem and its watershed, such as precipitation, land use, and lake morphology, will be more relevant in explaining large-scale spatial patterns of taxonomic and functional diversity of zooplankton compared to in-lake predictors like lake habitats and limnological characteristics.

## Methods

### Study area

**Fig. 1:**
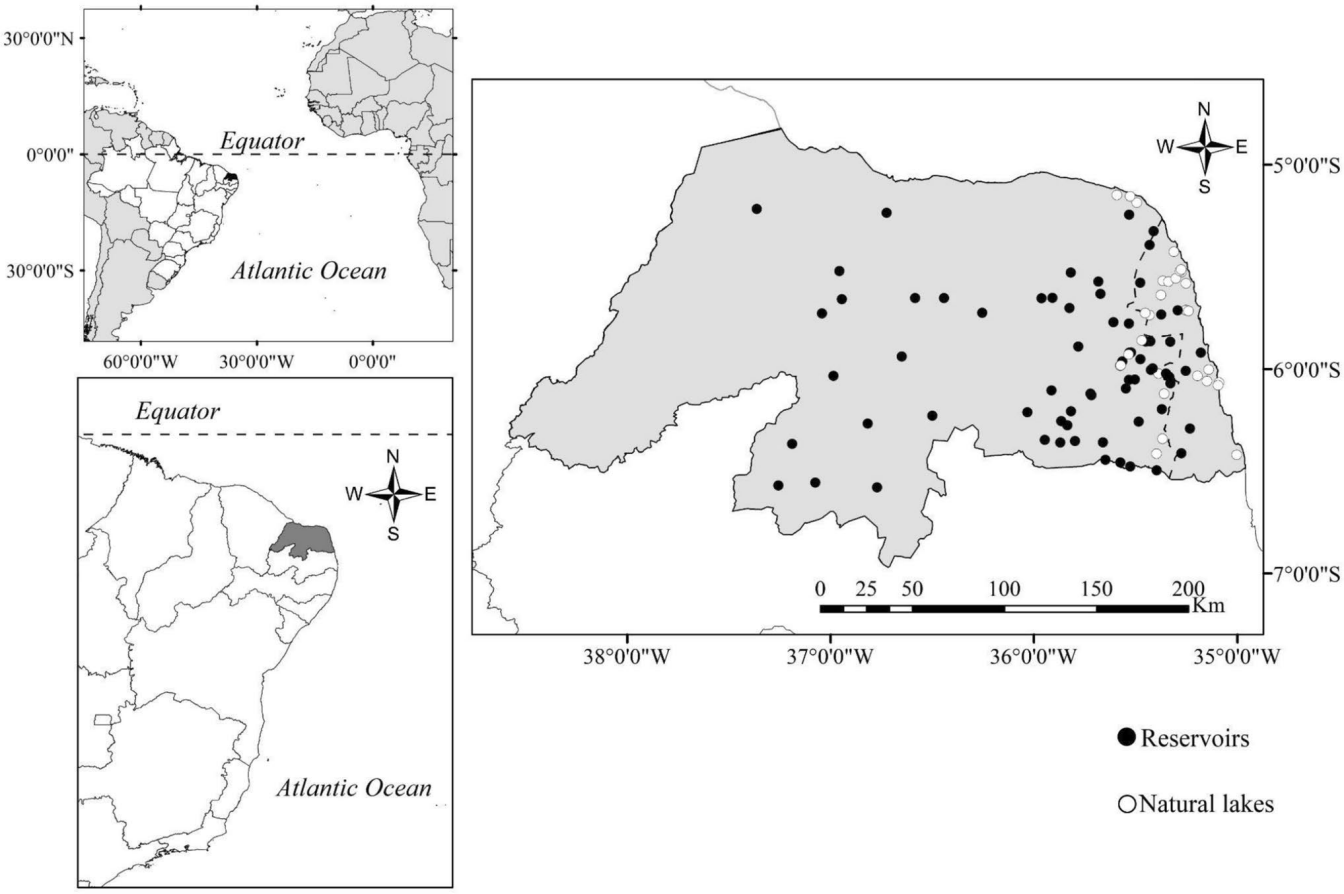
Geographical representation of the studied water bodies sampled on Rio Grande do Norte, Brazil. Natural lakes are represented by white dots and reservoirs by black dots. The dashed line depicts semi-arid delimitation. To the right of the line is the humid region and to the left is the semi-arid

This study was conducted across 98 lentic aquatic environments located in the state of Rio Grande do Norte, Northeast Brazil. Thirty ecosystems were natural lakes and 68 were reservoirs (hereafter referred to as lake origin; Fig. 1). Most (68%) of the ecosystems are in a semi-arid region, with annual accumulated precipitation ranging from 400 to 800 mm. The other environments are distributed along the coastal region, with annual accumulated precipitation ranging from 800 to 1200 mm (Fig. 1). The reservoirs have low flow rates and high-water residence times and are generally surrounded by poorly permeable soils. In contrast, natural lakes are mainly surrounded by sandy soils and are supplied by groundwater. The studied sites occupy 14 hydrographic basins and span an area of approximately 36,000 km^2^. Most ecosystems have a small surface area (89% < 1 km^2^) and low depth (90% < 4 m; see details in Nobre et al. (2020)). The environmental characteristics of the studied ecosystems include a wide gradient of catchment land use patterns and trophic status (see Cabral et al. (2020) and Nobre et al. (2020) for more details).

### Water sampling and analysis

In each lake, water samples were collected at six different points within two distinct habitats (hereafter referred to as habitat identity): three points randomly chosen in the littoral region among aquatic vegetation stands, and three points randomly chosen in the limnetic region, near the middle portion of the water body and away from aquatic vegetation. Since the water column of the sampled ecosystems is not stratified, we collected water samples near the surface (0.3 m depth) using a 2-L Van Dorn bottle. The three samples from each lake habitat were then mixed in a bucket, and a single 1-L integrated water sample per habitat was used for further limnological analysis. Samples were placed in acid-washed plastic bottles and frozen until laboratory analysis. The total sampling design comprised 196 samples: 98 from the littoral habitat and 98 from the limnetic habitat.

In the laboratory, we determined the values of chlorophyll-a (ChloA) according to the method proposed by Jespersen and Christoffersen (1987) and total phosphorus (TP) according to Murphy and Riley (1962). The values of total nitrogen (TN) were determined through an automatic TOC-V analyzer (Shimadzu) (see Nobre et al. (2020) for further details).

### Determination of lake morphometry, catchment land use properties, and precipitation patterns

Lake surface area (LA) and lake perimeter (LP) were estimated by outlining polygons (shapefiles) from satellite images using the ArcGIS 10.5 software (ESRI 2017). We estimated lake volume (LV) using the hyperbolic function 0.43 × LA × depth (Post et al. 2000; Nobre et al. 2020). Lake depth was measured at a midway point of each ecosystem using a calibrated rope. To quantify lake catchment’s properties, we first delimited the catchment limits for each ecosystem using ArcHydro 2.0 Toolbox (ArcHydroTools 2011) from ArcGIS 10.5 software (ESRI 2017). Then, we estimated the catchment area of lakes (CA). Furthermore, since previous studies have demonstrated that the distance of the land use practices to the aquatic system may contribute to different effects of land use on the aquatic ecosystems, we also delimited a buffer area (BU) around each ecosystem. The BU was estimated as an area around the lake comprising a 100-m wide strip of land starting from the lake shore. The BU delimitation seeks to detect the potential magnitude of allochthonous inputs derived from the proximal zone of the aquatic ecosystem and quantify the most direct impact of human occupation on the littoral zone of the lakes. We delimited CA and BU using the image processing tools of the ArcMap 10.5 software (ESRI 2017). Then, we used the catchment and lake morphometric variables to calculate catchment area-to-lake volume ratio (CA:LV), buffer area-to-lake volume ratio (BU:LV), and lake perimeter-to-lake volume ratio (LP:LV) for each ecosystem.

Land use properties of lake catchments were based on the data provided by the MapBiomas Project-Collection 3 of Brazilian Land Cover and Use Map Series (MapBiomasProject 2018). As land use properties, we estimated the extent of land use types and land use diversity at two spatial resolutions (i.e., for CA and BU). First, we classified land cover types into three major groups: natural areas, anthropic areas, and surface waters. Natural areas comprise four classes of land cover types, which preserve their pristine conditions (i.e., forest, savanna, wetlands, and grassland). Anthropic areas comprise classes of land cover corresponding to seven types of land use-associated human activities (i.e., pastures, cultivated areas, mosaics of agriculture and pastures, urban infrastructure, non-vegetated areas, mining, and aquaculture areas). Finally, surface waters comprise all aquatic environments such as rivers, lakes, and ponds occurring within the limits of the CA or BU, excluding the area corresponding to the lake itself. Then, using ArcGIS 10.5, we quantified the absolute and relative measures of land use extent for a given land use class for both the CA and BU. Absolute measures for each of the 12 classes of land use were estimated as the total area covered by a respective land use class within the limits of CA and BU. Relative extent of land use classes for CA and BU were finally calculated by dividing the absolute extent of a given land use category by the total area of the CA and BU, respectively. Data on the relative area of each land use class are available in the Supplementary Material (Table S1). However, for the simplicity of statistical analysis, we expressed land use extent for CA and BU as the total relative extent of anthropic areas within each spatial resolution, namely AntCA and AntBU, respectively. AntCA and AntBU corresponded to the sum of the relative cover areas of the seven classes of land use types related to human activities. To verify the potential effect of the input of allochthonous material from the terrestrial environment, considering the dilution capacity of the lake, we calculated the ratios of AntCA and AntBU in relation to the volume of the lake, namely AntACA:LV and AntABU:LV, respectively.

To quantify the influence of precipitation patterns, we calculated the magnitude (Totrain) and the variability (CVrain) of the precipitation for each water body. Totrain corresponds to the accumulated monthly precipitation from 2 years before samplings, spanning from September 2010 to September 2012. CVrain corresponds to the coefficient of variation of the monthly precipitation in the same period. We obtained this data from seven climatic stations distributed in the state of Rio Grande do Norte, and the final values of Totrain and CVrain correspond to the weighted average of the values for the different stations, with weights proportional to the distance between the water body and the climatic station (see Nobre et al. (2020) for details).

### Zooplankton samplings and taxonomic and functional diversity determination

We collected zooplankton just below the water surface (approximately 0.3 m deep) in both littoral and limnetic habitats using a 2-L Van Dorn bottle. Three samples were collected in each habitat, and the samples from each habitat were pooled in a 20-L bucket. Then, water was filtered through a plankton net (50 µm mesh size) to filter zooplankton. Filtered zooplankton samples were concentrated and immediately stored in 150-ml glass flasks with sugar formaldehyde solution (final concentration of 4%). We identified and quantified organisms in the laboratory until the species level using specialized literature (e.g., Koste 1978; Elmoor-Loreiro 1997). We quantified rotifers under a microscope using a Sedgewick-Rafter chamber and cladocerans and copepods under a stereomicroscope using a Bogorov chamber (see details in Cabral et al. (2020)). We performed the identification on three replicates per sample with at least 100 individuals of the most abundant organisms in each replicate, and we evaluated the samples entirely to search for rare species. Zooplankton species abundance was calculated by multiplying the average number of species’ individuals in the sample by the total volume of water filtered in the field.

We described the taxonomic and functional diversity of zooplankton community using Simpson diversity index (D) as taxonomic diversity response variable, and functional divergence (FDiv) as functional diversity response variables (Laliberté and Legendre 2010; Mouchet et al. 2010). The combined use of taxonomic and functional diversity measures has been widely recommended in the literature (Naeem and Wright 2003; Humbert and Dorigo 2005; Deosti et al. 2021; Dos Santos et al. 2025). Although taxonomic measures are important because they represent biological units, such measures do not provide information about how environmental characteristics affect the selection of traits present in a community. On the other hand, functional measures allow inferences about which traits are being selected as well as the role played by species in the structuring and functioning of ecosystems (Tilman et al. 1997; Covich et al. 2004; Mason and De Bello 2013). These indices are based on the species traits and express the functional differences between the species in a multidimensional space of characteristics (Mouchet et al. 2010). For example, a community that is taxonomically diverse but not functionally diverse may suggest high functional redundancy and indicate that factors such as competition are not important. On the other hand, high functional diversity may indicate that the environment selects species with the ability to explore distinct and complementary niches (Ellingsen et al. 2007; Rodrigues-Filho et al. 2023). FDiv is weighted by species relative abundances and, therefore, accounts for a greater weight for dominant species and functional traits within communities. FDiv analyzes how species traits are distributed within the functional space. Essentially, it examines the average distance of species from the centroid of the functional space, emphasizing how species are distributed around a central point. This measure is important for understanding how functional variation is organized and can indicate the presence of distinct functional niches within the community (Mason and De Bello 2013). For FDiv quantification, we used one continuous and five discrete functional traits as follows: body size (continuous), feeding mode (scraping, microphagous, raptorial, filtering, filtering-B – Bosminidae mode, filtering-D – Daphniidaemode, filtering-S – Sididae mode), food preference (herbivore, carnivore, omnivore, omni-herbivore, detritivore), type of reproduction (asexual/sexual, sexual), habitat (benthic/periphytic, periphytic, planktonic, planktonic/semi-planktonic, epiphytic/planktonic, planktonic/periphytic, benthic/periphytic/planktonic), and body defenses/appendices (carapace, lorica, exoskeleton, thin lorica, lorica spines, geltube, spines/body expansions). We determined the average body size of each species after measuring at least 30 individuals of each species following the method proposed by Ruttner-Kolisco (1977) for rotifers and Bottrell et al. (1976) for microcrustaceans. In the case of rare species, all individuals present in the sample were measured. Data on the functional traits of each species are available in the Supplementary Material (Table S2). We chose these traits because they adequately describe the multiple responses of zooplankton organisms to environmental conditions and have been widely used in studies on the functional diversity of zooplankton (Barnett et al. 2007; Obertegger et al. 2011; Litchman et al. 2013). FDiv was calculated using the Gower dissimilarity method modified by Pavoine et al. (2009). We used the maximum number of traits that allows the *S* ≥ *2^t* condition to be met, where *S* is the number of species and *t* is the number of traits. The indices were calculated through the dbFD function of the FD package (Laliberté and Legendre 2010) in the R environment.

### Statistical analysis

To verify the individual importance and potential interactions of in-lake and out-lake predictors on zooplankton Simpson and Fdiv, we used regression tree analysis (RTA). RTA is a non-parametric powerful analytic tool used to determine the most important variables in a particular dataset and explain the variation of a Breiman single response variable by one or more explanatory variables (Breiman et al. 1984). The advantage of RTA over other multivariate tests is that it can unravel interactions among predictors and produce a straightforward way to graphically visualize these interactions. When carrying out RTA, it is necessary to control the number of explanatory variables entering the model to avoid overfitting the data. In order to complement our approach and to maximize predictive accuracy, we also ran Random Forest analysis. Random Forest is a robust statistical approach for ecological data analysis, as it accommodates complex, non-linear relationships and interactions among predictors. It performs well with datasets containing both categorical and continuous variables, while being relatively resistant to overfitting. Additionally, it provides measures of variable importance, which are valuable for identifying key environmental drivers of ecological patterns.

All variables initially considered predictors of zooplankton diversity with their respective description, rationale, source, and measurement method can be found in Table S3 of the Supplementary Material. As some variables are correlated, we performed a previous selection. First, we applied pairwise Spearman’s correlations. For variables highly correlated (*r* ≥ 0.6), we excluded those with higher values of variance inflation factor (VIF) to avoid spurious correlations (Lepš and Smilaur 1998). Because TP, TN, and ChloA were all significantly correlated with each other and for better interpretability, we created a “trophic index” composite variable (TI) by running a Principal Component Analysis (PCA) on the three response variables (similarly to Knoll et al. (2015) and Nobre et al. (2020)). The first PCA axis explained 64% of the variance among the studied freshwater systems, and thus, the scores were used as our TI composite variable. The PCA was performed in R (R Core Team 2018) using the vegan package (Oksanen et al. 2018) and the biplot can be seen in the supplementary material (Fig. S1). After this preliminary selection, we considered Totrain, AntBU, AntCA, LP:LV, AntABU:LV, AntACA:LV, and TI as continuous predictors, and habitat identity, macrophyte presence, and lake origin as categorical predictors in RTA. We chose these variables because they were related to zooplankton diversity in previous studies (Porcel et al. 2020; Setubal et al. 2020; Deosti et al. 2021; Diniz et al. 2021), and the rationale for including these parameters on the model can be found in Table 1. Correlation and VIF analysis were conducted with R using the stats (R Core Team 2018) and *usdm* (Naimi 2015) packages, respectively.

**Table 1:** Mean, maximum and minimum values, and standard deviation (SD) of the variables used in the regression tree analysis model with their respective description, rationale, source, and measurement method

Variables	Units	Rationale	Source	Measurement method	Mean ± SD (min, max)
*Response variable*					
Simpson diversity (*D*)		Diversity measure, which considers the number of species present, as well as the relative abundance of each species. Often used to quantify the biodiversity of an ecosystem	Our survey	The index was calculated as follows: *D* = Σ*n*_i_(*n*_i_−1)/*N*(*N*−1) where *n* = the total number of organisms of a particular species; *N* = the total number of organisms of all species	0.531 ± 0.291 (0.0003, 0.935)
Functional divergence (FDiv)		FDiv represents the degree to which the abundance of a community is distributed toward the extremities of occupied trait space. FDiv is expected to increase when niche complementarity enhances the relative abundances of species. FDiv has most often been linked to community assembly processes and ecosystem functioning (Mouchet et al. 2010; Mason and De Bello 2013)	Our survey	The index was calculated in the R environment through the dbFD function of the FD package (Laliberté and Legendre 2010)	0.346 ± 0.14 (0.022, 0.741)
*Explanatory variables*					
Trophic index (TI)		Proxy of ecosystem productivity	Our survey and R version 4.2.1	The Trophic index was obtained through a Principal Component Analysis (PCA) of water quality variables (TN, TP and Chl-a). PCA was performed in R v 4.2.1 using the Vegan package	0.00 ± 1.386 (−1.860, 6.291)
Total precipitation (Totrain)	mm	Serves as a proxy for the potential input of allochthonous materials, such as nutrients and sediments, into aquatic systems. These materials are transported through subsurface and groundwater flow. Additionally, precipitation influences the dilution capacity of the aquatic system	INMET	Monthly cumulative precipitation from September 2010 to September 2012	4.58 ± 6.42 (0.26, 30.68)
Lake perimeter to volume ratio (LP:LV)	m	Represents the potential magnitude of connectivity, by volume unit, between the aquatic system and the adjacent terrestrial ecosystem	Our survey and TOPODATA	Lake’s polygon shapefiles (ArcGis 10.5.1—ESRI 2017) to obtain area and perimeter, field survey to obtain lake depth. Lake volume was calculated from the hyperbolic function 0.43 × lake area × depth (Post et al. 2000)	0.04 ± 0.05 (0.003, 0.24)
Land use (anthropogenic area)	%	Represent the magnitude and quality of allochthonous material entering aquatic ecosystems. It considers the type of land use, proximity use (buffer or catchment area) and extension of land use (total area of catchment and buffer)	Mapbiomas 3.0	Land use classification for Caatinga and Atlantic Forest biomes. Agriculture, pasture and urban/developed uses were grouped into anthropogenic use. Lakes, catchments, and buffer areas were calculated from polygon shapefiles (ArcHydro 2.0 Toolbox – ArcHydro 2011—on ArcGis 10.5.1—ESRI2017)	Buffer (AntBU): 60 ± 27 (0.1, 99) Catchment (AntCA): 62 ± 26 (0.1, 99)
Land use (anthropogenic use) to volume ratio		Represent the level of connectivity and the potential magnitude of the input of allochthonous material from the terrestrial environment considering the volume of the lake. It considers the type of land use, proximity use (buffer or catchment area) and extension of land use (total area of catchment and buffer)	Our survey, TOPODATA and Mapbiomas	Total area (km^2^) of anthropogenic use in catchment or buffer calculated was mentioned above. Lake volume was calculated from the hyperbolic function 0.43 × lake area × depth (Post et al. 2000)	Buffer (AntABU:LV) 2.870 ± 4.575 (0, 21.457) Catchment (AntACA:LV) 385.639 ± 1691.736 (0, 14,661.9)
Lake origin		The geomorphological origin and hydrodynamic characteristics of several types of ecosystems may have important carry-over effects on their resident aquatic communities	Our survey	Natural lakes or reservoirs. Reservoirs were classified as artificial ecosystems since they are man-made buildings	
Habitat identity		The type and structural complexity of lake habitats and the horizontal compartmentalization creates distinct environmental conditions within a single ecosystem affecting planktonic communities	Our survey	Littoral or limnetic. The littoral region of the lake consists of the transition area and is in direct contact with the terrestrial environment. The limnetic region consists of the middle area of the aquatic environment	
Macrophytes presence		Provide shelter from predators, offer surfaces for food resources (like periphyton and detritus), and create more stable environmental conditions	Our survey	Presence or absence of any type of macrophytes	

We performed one RTA for D and one for FDiv using the *rpart* package in R (Therneau et al. 2015). The regression tree was built by considering the optimization of the hyperparameters minimum number of splits (minsplit) and the complexity parameter (cp). We applied the leave-one-out cross-validation (LOOCV) to choose both minsplit and cp. In the result section, we report the *R*^2^ (*R*^2^ = 1 – relative error), which represents the percentage of variation in the dataset explained by our model and the RMSE, the square root of the variance of the residuals, which indicates the absolute fit of the model. The lower values of RMSE represent a better fit. The figures for each node were made using the software GraphPad Prism version 8.0, and the final RTA figures were made using the software Inkscape vector graphics editor. We also applied Random Forest (RF) models to evaluate the effects of multiple environmental variables on zooplankton diversity metrics. RF models were fitted with 1000 trees, and variable importance was quantified using permutation-based measures of mean squared error increase. To ensure robust assessment of model performance given the relatively small sample size (*n* = 98 water bodies), we also adopted leave-one-out cross-validation (LOOCV) as the validation strategy. Model performance was summarized by the percentage of variance explained, coefficient of determination (*R*^2^), and root mean squared error (RMSE). Variable importance was assessed using permutation importance scores and visualized through importance plots. We performed the RF using the *randomForest* package in R.

## Results

We identified 188 zooplankton species across the water bodies evaluated in this study. Rotifera was the most diverse group, with 111 species, followed by Cladocera with 47 species, and Copepoda with 30 species. Considering lake origin and habitat identity, we found higher values for species richness, D, and FDiv in the littoral zones of natural lakes (Table 2).

**Table 2:** Mean values and standard deviation (SD) of the main variables evaluated in this study for artificial and natural water bodies and for limnetic and littoral habitats

Variables	Artificial				Natural			
	Limnetic		Littoral		Limnetic		Littoral	
	Mean	SD	Mean	SD	Mean	SD	Mean	SD
Chlorophyll a (µg L^−1^)	80.231	124.497	96.976	170.703	19.621	59.503	30.282	65.227
Total phosphorus (mg L^−1^)	0.290	0.232	0.306	0.237	0.129	0.085	0.132	0.101
Total nitrogen (mg L^−1^)	5.768	2.298	5.696	2.366	3.712	1.841	3.741	1.655
Richness	10.914	5.554	11.655	6.571	13.625	5.471	17.100	8.617
Simpson diversity	0.474	0.305	0.473	0.325	0.578	0.227	0.649	0.229
Functional divergence	0.326	0.148	0.331	0.151	0.401	0.131	0.404	0.124

With respect to landscape characteristics related to land use, we observed considerable variability in use types (Supplementary Material, Table S1). On average, about 60% of both CA and BU consisted of anthropogenic areas, predominantly farmlands such as pastures and cultivated fields. Natural lakes, on average, had the highest proportion of anthropized areas within their CA. Examining morphometric characteristics, we found that reservoirs generally exhibited higher average values for surface area, LV, CA, and the CA:LV ratio compared to natural lakes. Conversely, natural lakes had a higher average Totrain. For limnological variables, reservoirs displayed the highest average values for ChloA, TP, and TN (Table 2). Overall, we observed no differences in the values of limnological variables between the littoral and limnetic habitats in either reservoirs or natural lakes, except for ChloA, where we found higher concentrations in the littoral zones of both natural lakes and reservoirs (Table 2). Although the abundance and identity of macrophytes were not quantified, our study recorded the presence and absence of aquatic vegetation. We found that 100% of the natural lakes sampled had submerged macrophytes.

**Fig. 2:**
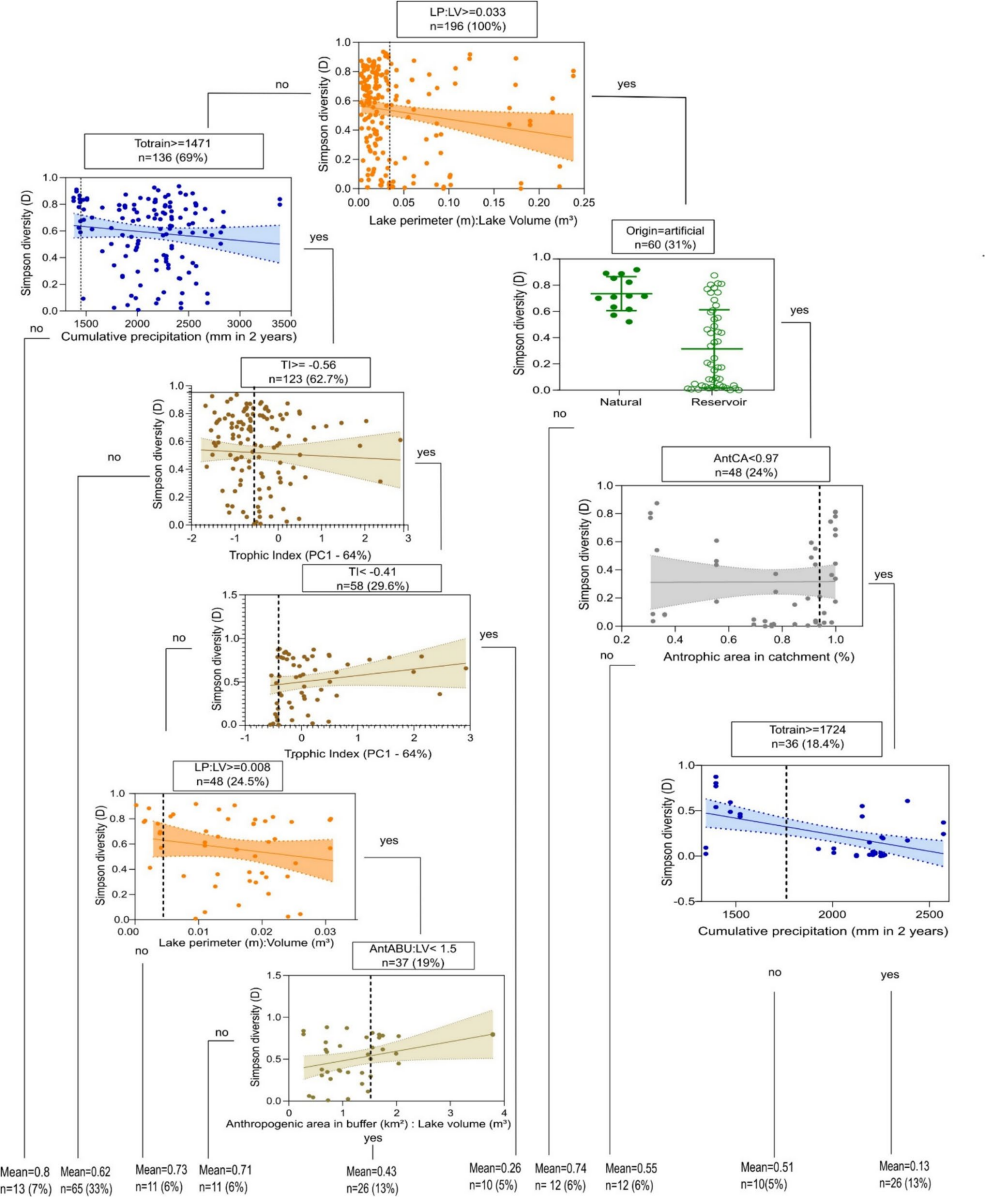
Regression tree showing the relationships between Simpson diversity index (*D*) of zooplankton community and limnological, climatic, landscape, and land use properties for the studied lakes. TI is a composite response variable originated from the PCA using TN, TP, and ChloA. Each split in the tree represents a Yes or No answer to the condition stated in each node box. The relationship between the response variable and the selected variable at each node is represented by correlation graphs. LP:LV = lake perimeter-to-lake volume ratio, Totrain = accumulated monthly precipitation over 2 years, from September 2010 to September 2012, TI, trophic index; AntCA, % of anthropogenic land use on the catchment area extent; AntABU:LV, total area (km^2^) of anthropogenic use in buffer area-to-lake volume ratio

According to the results of the RTA, seven variables significantly contributed to explaining the variation in zooplankton Simpson’s diversity: LP:LV, Totrain, lake origin, AntCA, AntABU:LV, and TI. This model accounted for 47% of the variance (*R*^2^ = 0.47) and had an RMSE of 0.21. In the first split node of the tree, we observed a marked reduction in zooplankton taxonomic diversity as the LP:LV ratio increased (Fig. 2). Specifically, environments with an LP:LV ratio greater than 0.033 tended to exhibit lower Simpson’s diversity values. Within these environments, reservoirs hosted zooplankton communities that were significantly less taxonomically diverse than those in natural lakes (right branch of the tree – Fig. 2). Among reservoirs where AntCA was less than 97% and Totrain was 1724 mm or lower, we observed higher Simpson’s diversity compared to reservoirs with higher Totrain values. The influence of Totrain on zooplankton taxonomic diversity was also evident in lakes with LP:LV ratio below 0.033 (left branch of the tree – Fig. 2). There was a noticeable reduction in Simpson’s diversity for lakes with Totrain exceeding 1471 mm. Lakes with the highest TI values displayed lower zooplankton taxonomic diversity, especially those with higher LP:LV ratio and AntABU:LV (Fig. 2). Considering the RF results, the model for taxonomic diversity presented RMSE = 0.226, *R*^2^ = 0.395, and the most important variables were Totrain, AntCA, AntACA:LV, LP:LV, AntBU, AntABU:LV, TI, and origin. The graph showing the importance of the variables is found in the supplementary material (Fig. S2).

**Fig. 3:**
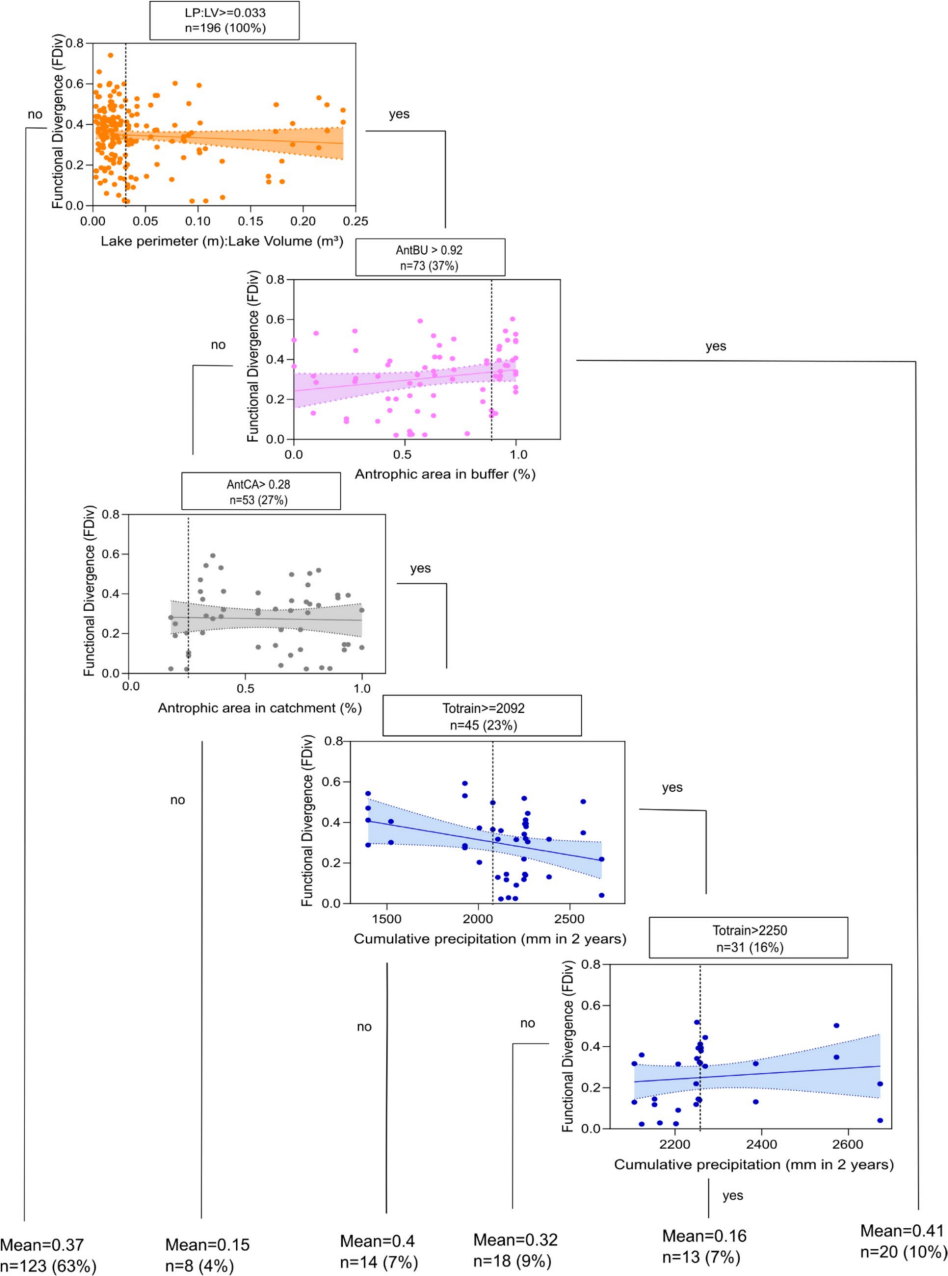
Regression tree showing the relationships between functional divergence of zooplankton community and limnological, climatic, landscape, and land use properties for the studied lakes. Each split in the tree represents a Yes or No answer to the condition stated in each node box. The relationship between the response variable and the selected variable at each node is represented by correlation graphs. LP:LV, lake perimeter-to-lake volume ratio; Totrain, accumulated monthly precipitation over 2 years, from September 2010 to September 2012; AntCA, % of anthropogenic land use on the catchment area extent; AntBU, % of anthropogenic land use on the buffer area extent

For functional diversity, the RTA model identified four key variables: LP:LV ratio, AntBU, AntCA, and Totrain. This model presented *R*^2^ = 0.03 and had an RMSE = 0.147. Similarly to what was observed for taxonomic diversity, LP:LV ratio was the primary split node in the FDiv tree, with the same threshold value (Fig. 3). Lakes with an LP:LV ratio above 0.03 generally exhibited lower FDiv values. However, AntBU values greater than 0.92 appeared to mitigate the negative effects of a high LP:LV ratio, as lakes with a high proportion of anthropic shoreline use tended to show higher FDiv values. In lakes where AntBU was below 0.92 and AntCA exceeded 0.28, Totrain became a major influencing factor. Specifically, lakes with less than 92% anthropic area around their shores but more than 28% in the remaining catchment area tended to have lower zooplankton FDiv under high precipitation conditions (Totrain > 2092 mm). Finally, those lakes with Totrain values between 2092 and 2250 mm exhibited the lowest FDiv values. The results of the RF model for functional diversity presented RMSE = 0.144, *R*^2^ = 0.037, and the most relevant predictor variables were LP:VL, AntBU, AntABU:LV, Totrain, TI, AntCA, and AntACA:LV. The graph showing the importance of the variables is found in the supplementary material (Fig. S2).

## Discussion

Our results demonstrate that different aspects of zooplankton biodiversity are influenced by the interplay of different predictors constituting lakes’s waterscape. We observe that although a multitude of proximate and ultimate factors operating at different temporal and spatial scales can influence zooplankton diversity across the catchment-to-lake waterscape continuum (Karpouzoglou and Vij 2017; O’Sullivan et al. 2022), the out-lake predictors, especially those related to lake morphometry, climatic conditions, landscape variables, and anthropogenic activities in the catchment and buffer areas, were most relevant. Consistent with our hypothesis, out-lake predictors were more important in determining both the taxonomic and functional diversity of zooplankton. While limnological characteristics were also included in our model for taxonomic diversity, the effects of these in-lake predictors were mediated by larger-scale factors, resulting in a cascade of nested effects. We found that out-lake predictors either directly influenced diversity or interactively mediated the magnitude of the effects of allochthonous inputs on local characteristics such as limnological conditions.

In fact, for functional diversity, only out-lake predictors were selected in the model. However, lake origin and in-lake features such as limnological parameters remain important for understanding the taxonomic diversity of zooplankton communities. High connectivity and susceptibility of environments to external inputs (out-lake predictors) generally lead to greater input of allochthonous material into the lakes (Staehr et al. 2012) and changes in limnological parameters (in-lake predictors) promoting a cascade of effects. This, in turn, could have negatively impacted both taxonomic and functional diversity in our study. Although functional diversity indices are commonly used to assess the productivity of ecological systems (see Setubal et al. (2020)), in this study indirectly assessed by the trophic index, we observed that FDiv was more sensitive to variations in external factors. However, we emphasize that the indirect mechanisms by which external factors affect aquatic communities involve modifications in limnological conditions (in-lake factors) and can be highly intricate and potentially non-linear. Our results suggest that, although biodiversity responses to different environmental predictors can be extremely complex, ecosystem metrics such as functional diversity, tend to respond better to predictors that operate at larger scales, while metrics that are more sensitive to variations in species composition and density, such as taxonomic diversity, respond better to both proximate and ultimate predictors.

Contrary to our initial expectations, the model for functional diversity demonstrated lower explanatory power than the model for taxonomic diversity. The use of functional diversity metrics has been widely recommended in the literature for their ability to capture various aspects of biodiversity variation (Litchman et al. 2013; Laureto et al. 2015). However, quantifying functional diversity is highly dependent on the selection of functional traits, which, for some groups, still lack resolution and precise definition. For zooplankton, many functional traits are determined at broad taxonomic levels, such as family or order, leading to a strong taxonomic bias and an underestimation of specific variable characteristics within the same genus (Branco et al. 2023). Furthermore, most traits are categorical, and much of the information regarding metabolic and physiological rates remains unknown (Barnett et al. 2007). As a result, functional measures can inflate community redundancy because, due to the low resolution of functional traits, many species are functionally similar. This limits the ability of functional indices to detect subtle variations in diversity and leads to models with lower explanatory power (Schleuter et al. 2010). Therefore, studies that evaluate both taxonomic and functional aspects of communities have the potential to capture different facets of biodiversity in a complementary manner and are widely recommended (Branco et al. 2023; Dos Santos et al. 2025).

The lake perimeter-to-volume ratio was one of the most relevant factors influencing the taxonomic and functional structure of the zooplankton community. This characteristic is closely related to the susceptibility of shallow lakes to material inputs from surrounding areas and their drainage basins (Doubek and Carey 2017; Porcel et al. 2020). A larger perimeter increases the transition zone between aquatic and terrestrial environments, facilitating the influx of allochthonous organic matter, nutrients, and pollutants from the catchment (Foley et al. 2005; Vanni et al. 2005; Jeppesen et al. 2009). In shallow systems, the effects of precipitation are particularly pronounced: rainfall events not only intensify the loading of external materials but also directly alter lake volume and water level due to the small storage capacity of these systems. Since volume acts as an indicator of the lake’s dilution and buffering capacity (Moses et al. 2011; Jeppesen et al. 2015; Winslow et al. 2015), the relatively small water volumes of shallow lakes make them more sensitive to fluctuations driven by precipitation. Consequently, lakes with a high LP:LV ratio combine greater susceptibility to external disturbances with limited capacity to dilute them, meaning that small, shallow, and irregularly shaped lakes are strongly affected by land use changes and hydrological variability in their catchment areas. Our results show that the LP:LV ratio is a significant factor affecting zooplankton taxonomic and functional diversities because it modulates the magnitude of the impacts of other environmental predictors. Even if a lake’s surrounding area and catchment basin are largely converted to anthropogenic use, a low LP:LV ratio (indicating deep lakes with a reduced water-land transition zone) means the environment will be less susceptible to external inputs due to reduced connectivity and greater dilution capacity.

Although many studies have highlighted the importance of lake size and shape for various ecological processes (Lacroix et al. 1999; Winslow et al. 2015; Dos Santos et al. 2025), water quality (Nielsen et al. 2012; Nobre et al. 2020), and the development of marginal vegetation (Kolada 2014), few have investigated how the lake perimeter-to-lake volume ratio modulates the effects of land use in the surrounding area and catchment on aquatic communities, especially zooplankton. Contrasting results have been observed regarding the impact of land use on zooplankton communities. Some studies show reduced taxonomic and functional diversity in lakes with highly anthropized catchments (Dodson et al. 2007; Cortez-Silva et al. 2020; Sługocki et al. 2024), while others find no such effects (Meier et al. 2015; Rocha et al. 2020; Popa et al. 2023). These discrepancies may be due to the varying susceptibility of lakes, as indicated by the LP:LV ratio, which can either enhance or neutralize the impact of land use. Our study, when considering the LP:LV ratio, allows us to understand the possible modulating importance of lake morphometry on the impacts of land use and, consequently, on lake biodiversity. In addition to lake morphometry, climatic characteristics such as precipitation also influence the effects of land use. Our results indicate that accumulated precipitation is a significant factor affecting both the taxonomic and functional diversity of the zooplankton community. While lake morphometry affects the degree of susceptibility and resilience to allochthonous material inputs, precipitation influences the intensity and frequency of these inputs (Carpenter et al. 2018). Consequently, even with significant environmental changes in lake surroundings, the impact of surface runoff and other input pathways of allochthonous materials and pollutants through subterranean aquatic connections is largely determined by the water balance between precipitation and evaporation (Jeppesen et al. 2009; Carpenter et al. 2018; Wang et al. 2018). Thus, both internal and external lake variables are crucial for understanding their interactive effects with broader-scale factors like land use.

Although the shape of the lake and precipitation do not directly affect the zooplankton community, these variables influence the magnitude of the effects of in-lake factors. For example, limnological variables intrinsically related to habitat quality are known to be affected by the input of external materials, particularly nutrients, through surface runoff (Staehr et al. 2012; Carpenter et al. 2018). Our results indicate high trophic index values negatively impact the taxonomic diversity of zooplankton in lakes with high external input (high Totrain values). These variables are strongly associated with the eutrophication process, which has been previously reported to the environments studied (Junger et al. 2019; Nobre et al. 2020). Additionally, the negative effects of trophic level on taxonomic diversity are linked to a decrease in species that are most sensitive to changes in water quality. This shift in environmental conditions has also promoted the selection of species with traits better suited to surviving under eutrophic conditions. Josué et al. (2019) found that eutrophication and the resulting increase in toxic algae were responsible for reductions in zooplankton functional and taxonomic diversity.

In lakes with high LP:LV ratios, we observed lower taxonomic diversity in reservoirs. On average, artificial water bodies have larger surface areas, volumes, CA and LP:LV ratios compared to natural water bodies, as they are primarily constructed for human use. Reservoirs in the studied region were created recently (within the last 50 to 100 years) to meet human needs such as water provision, irrigation, and energy production (Rosenberg et al. 2000; Havel et al. 2005). In contrast, natural lakes, formed through natural geomorphological processes (Havel et al. 2005), exhibit greater variability in land use within their catchment areas compared to reservoirs. Many natural lakes are situated in the coastal region of the Atlantic Forest biome, where their catchment areas often include fragments of native vegetation. Several studies suggest that diversity tends to be higher in natural environments due to their more complex and heterogeneous habitats (McKindsey and Bourget 2001; Dodson et al. 2007; Heino et al. 2021). Our finding of greater taxonomic diversity in natural lakes supports this idea. Furthermore, since natural lakes are older than reservoirs, it is expected that their diversity would be higher. This expectation arises from the longer time available for regional dispersion processes to act (Dodson et al. 2007). However, in the environments studied here, we believe that the effects of the environment’s origin are more closely related to the greater impacts experienced by reservoirs, regardless of their age.

In general, we observed an elevated level of human activity in the catchment areas of both natural lakes and reservoirs. For the environments studied here, these activities primarily include urban infrastructure, subsistence agriculture, and the cultivation of fruit and sugar cane (Ribeiro et al. 2015). This region has the highest population density in the world under semi-arid conditions (Buainain and Garcia 2013) and the local population relies heavily on environmental resources for subsistence (Gariglio et al. 2010). These conditions have historically shaped the pattern of land use in the region, resulting in a high degree of human activity around lakes. Intense anthropogenic activity is recognized as a primary determinant of water quality and contributes to eutrophication in the artificial lakes studied here (Cabral et al. 2020; Nobre et al. 2020). Alfonso et al. (2010) found similar results in environments affected by anthropogenic changes and noted that these impacts might obscure the effects of ecosystem origin. Consequently, lower taxonomic diversity in artificial lakes is linked to the eutrophication process, which can have significant effects on biodiversity (Knoll et al. 2015; De Melo et al. 2019).

For zooplankton functional diversity, both the LP:LV ratio and Totrain, as well as out-lake variables related to land use, were included in the regression tree model. The lowest functional diversity (FDiv) values were observed in lakes with significant conversion of surrounding areas and high precipitation. Since a substantial portion of the catchment basin is affected by potentially polluting human activities, higher rainfall intensifies the diffuse input of nutrients (Dodson et al. 2005; Burgin et al. 2016; Gozlan et al. 2019). The reduction in functional divergence with increased anthropogenic activity in the catchment basin may be related to the loss of rare and complementary species. FDiv is sensitive to changes in community trait composition, and a decrease in FDiv indicates a reduction in the abundance of functionally complementary species (Mason et al. 2005; Mouchet et al. 2010). Thus, the loss of functionally distinct species and the increase in redundant species contribute to the impoverishment of community functions. Our results indicate a process of functional homogenization in the communities, with the extent of this effect being interactively mediated by out-lake (LP:LV ratio and Totrain) predictors.

The observed functional change was primarily driven by a reduction in functional traits associated with microcrustaceans (cladocerans and copepods) and an increase in traits related to rotifers. Previous studies have shown that cladocerans are more sensitive to changes in water quality and habitat structure (Suikkanen et al. 2013; Gutierrez et al. 2018). In contrast, rotifers are often the most species-rich group in aquatic environments undergoing eutrophication and environmental changes (Obertegger et al. 2011). Due to their short life cycle, generalist diet, and small body size, rotifers tend to become dominant in conditions of low competitive pressure from other zooplankton groups (Duggan et al. 2001). The presence of small-sized zooplankton may directly affect nutrient cycles and energy flows through primary producers to the other trophic levels, especially under eutrophication conditions (Josué et al. 2019). Therefore, the effects of external factors on the functional divergence of zooplankton, through the replacement of larger species, can promote modifications in trophic relationships and, ultimately, productivity and respiration rates (Hebert et al. 2017). Although it was not possible to measure in-lake variables related to zooplankton trophic interactions, in a previous study, Cabral et al. (2020) argue that the omnipresence of the omnivorous habit of fish in tropical environments (Jeppesen et al. 2010) could reduce the dependence and, consequently, the strength of interaction of fish with zooplankton in the environments studied. The omnivorous habit allows fish to explore different resources, including food of terrestrial origin, in such a way that it would decrease the predation rate on larger zooplankton. However, due to the lack of data on fish composition and the imbalance in the number of in-lake and out-lake variables, these inferences should be interpreted with caution.

Our results suggest that both in-lake and out-lake factors influence limnological characteristics, which act as ecological filters limiting the occurrence of species sensitive to habitat quality changes. This loss in taxonomic diversity led to reduced functional divergence and increased dominance. Additionally, reductions in functional diversity in environments with intensely occupied surrounding areas have been linked to decreased habitat complexity. For example, Andrade et al. (2022) observed that the occurrence of natural forests around the lake promotes an increase in functional diversity due to the greater variability of niches that a structurally more complex environment provides (greater input of gains and leaves from riparian vegetation). It has also been shown that urban and agricultural landscapes are strongly associated with reduced diversity (Shen et al. 2021; Andrade et al. 2022; Paquette et al. 2022; Sługocki et al. 2024; Flores-Mendez et al. 2024). However, research evaluating the effects of land use on both taxonomic and functional diversity is limited (Dos Santos et al. 2025).

## Conclusions

Through a broad spatial analysis of aquatic environments with different origins and morphologies, we observed that biodiversity responses to multiple in-lake and out-lake factors can be complex. Functional diversity measures were effective in detecting the effects of ultimate factors that operate at larger scales, especially out-lake factors. Taxonomic diversity measures were relevant in detecting the interactive effects of both out-lake and in-lake factors, since the former determined the magnitude of the effects of the latter. Our results indicate that factors such as lake morphology and rainfall intensity play a crucial role in determining the functional and taxonomic structure of zooplankton communities. These factors can also amplify the negative effects of converting natural areas into anthropogenic ones on water quality. Lakes that are more susceptible to allochthonous inputs of nutrients and pollutants due to greater connectivity (extensive water-land contact area) and surface runoff (intense rainfall) may experience community impoverishment and functional homogenization, as they support fewer species capable of surviving in conditions of low environmental quality. Therefore, using both in-lake and out-lake predictors, such as lake perimeter-to-volume ratio, precipitation, and the proportion of anthropogenic activities in the catchment area of both natural lakes and reservoirs, can serve as an effective and integrative proxy for several factors influencing aquatic community diversity.

## Supplementary Information

Below is the link to the electronic supplementary material.

ESM1 (DOCX 377 KB)

## Data Availability

Data will be made available on reasonable request.
